# Supplementary material for: The interactome of intact mitochondria by cross-linking mass spectrometry provides evidence for coexisting respiratory supercomplexes
Source: Mol Cell Proteomics. 2017 Dec 8;17(2):216–32. doi: 10.1074/mcp.RA117.000470 (PMC5795388; doi:10.1074/mcp.RA117.000470)
Supplement: Supplemental Data [file supp_RA117.000470_133922_0_supp_23978_fzffwf.zip › spectra_annotation/mito_spectra_annotation/116-2-4-1-20-1.pdf]

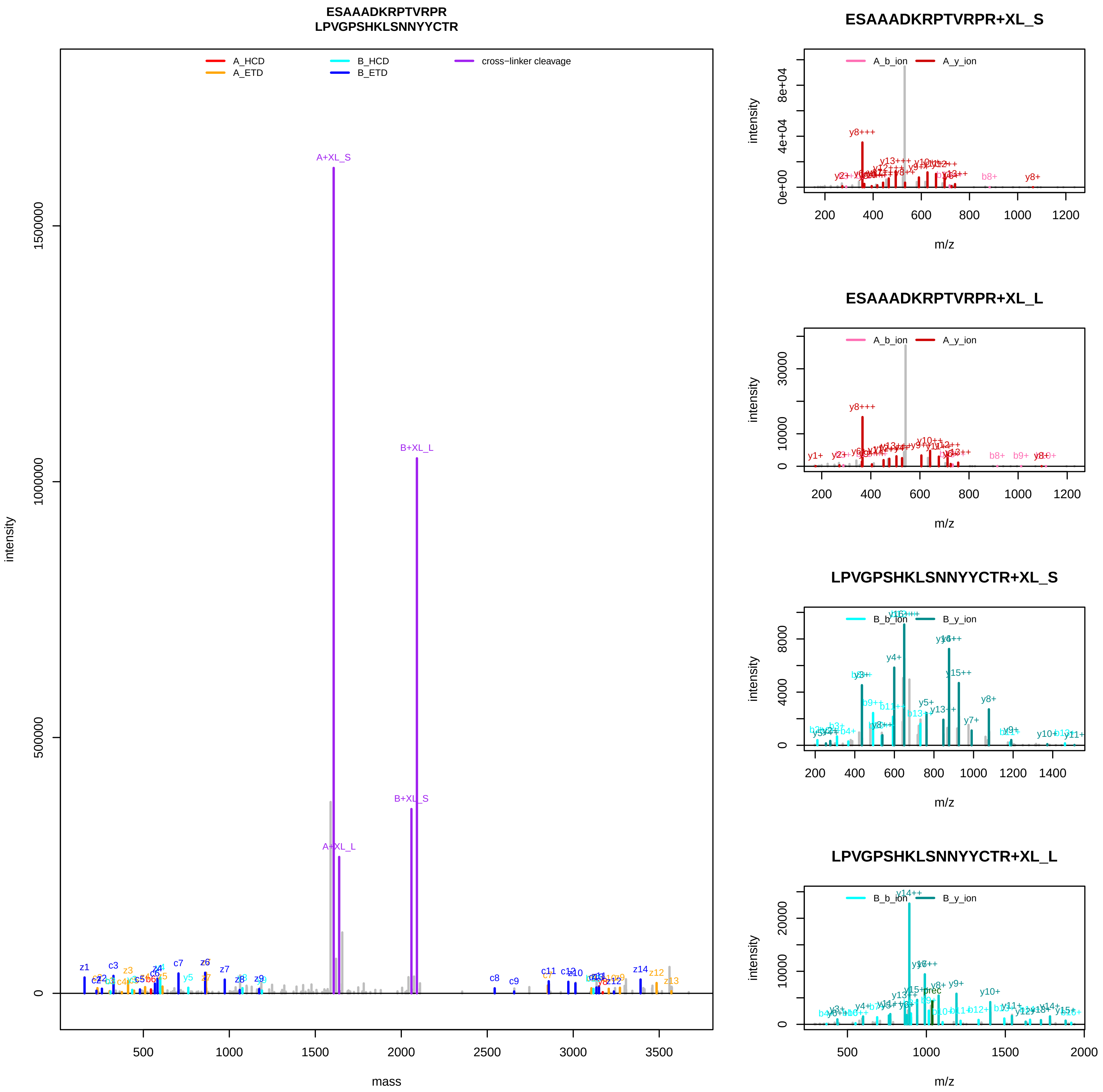
ESAAADKRPTVRPR
LPVGPSHKLSNNYYCTR
A_HCD
A_ETD
B_HCD
B_ETD
cross−linker cleavage
A+XL_S
B+XL_L
B+XL_S
A+XL_L
intensity
mass
1500000
1000000
500000
0
500
1000
1500
2000
2500
3000
3500
ESAAADKRPTVRPR+XL_S
A_b_ion
A_y_ion
y8+++
y8+
b8+
8e+04
4e+04
0e+00
m/z
ESAAADKRPTVRPR+XL_L
30000
20000
10000
LPVGPSHKLSNNYYCTR+XL_S
B_b_ion
B_y_ion
y4+
y15++
y8+
8000
4000
LPVGPSHKLSNNYYCTR+XL_L
y14++
y10+
prec
1400
1200
1000
800
600
400
200
